# Detection of *K-Ras* mutations in tumour samples of patients with non-small cell lung cancer using PNA-mediated PCR clamping

**DOI:** 10.1038/sj.bjc.6604925

**Published:** 2009-03-17

**Authors:** M Beau-Faller, M Legrain, A-C Voegeli, E Guérin, T Lavaux, A-M Ruppert, A Neuville, G Massard, J-M Wihlm, E Quoix, P Oudet, M P Gaub

**Affiliations:** 1 Laboratoire de Biochimie et de Biologie Moléculaire, Hôpital de Hautepierre, Centre Hospitalier Universitaire de Strasbourg, 67098 Strasbourg Cedex, France; 2 UMR7104, Institut de Génétique et de Biologie Moléculaire (IGBMC), 67400 Illkirch-Graffenstaden, France; 3 Pôle de Pathologie Thoracique, Service de Pneumologie, Nouvel Hôpital Civil, Centre Hospitalier Universitaire de Strasbourg, 67091 Strasbourg Cedex, France; 4 Pôle de Biologie, Laboratoire de Pathologie, Hôpital de Hautepierre, Centre Hospitalier Universitaire de Strasbourg, 67098 Strasbourg Cedex, France; 5 Pôle de Pathologie Thoracique, Service de Chirurgie Thoracique, Nouvel Hôpital Civil, Centre Hospitalier Universitaire de Strasbourg, 67091 Strasbourg Cedex, France

**Keywords:** non-small cell lung cancer, *K-Ras* mutations, sensitivity, real-time PCR, PNA

## Abstract

Non-small cell lung cancers (NSCLC), in particular adenocarcinoma, are often mixed with normal cells. Therefore, low sensitivity of direct sequencing used for *K-Ras* mutation analysis could be inadequate in some cases. Our study focused on the possibility to increase the detection of *K-Ras* mutations in cases of low tumour cellularity. Besides direct sequencing, we used wild-type hybridisation probes and peptide-nucleic-acid (PNA)-mediated PCR clamping to detect mutations at codons 12 and 13, in 114 routine consecutive NSCLC frozen surgical tumours untreated by targeted drugs. The sensitivity of the analysis without or with PNA was 10 and 1% of tumour DNA, respectively. Direct sequencing revealed *K-Ras* mutations in 11 out of 114 tumours (10%). Using PNA-mediated PCR clamping, 10 additional cases of *K-Ras* mutations were detected (21 out of 114, 18%, *P*<0.005), among which five in samples with low tumour cellularity. In adenocarcinoma, *K-Ras* mutation frequency increased from 7 out of 55 (13%) by direct sequencing to 15 out of 55 (27%) by clamped-PCR (*P*<0.005). *K-Ras* mutations detected by these sensitive techniques lost its prognostic value. In conclusion, a rapid and sensitive PCR-clamping test avoiding macro or micro dissection could be proposed in routine analysis especially for NSCLC samples with low percentage of tumour cells such as bronchial biopsies or after neoadjuvant chemotherapy.

Malignant transformations are the result of an accumulation of carcinogenesis steps corresponding to activation of oncogenes and inactivation of tumour suppressor genes (Bishop, 1991). Among the available candidates, the *K-Ras* proto-oncogene is the most well-studied cellular gene whose alterations seem to have an important role in the pathogenesis of human cancer. *K-Ras* oncogene is a known downstream signaling molecule in the EFGR-signaling pathway. *K-Ras* gene encodes a 21 kDa GTP-binding protein, which controls the mechanisms of cell growth and differentiation. Point mutations in the *K-Ras* gene lead to uncontrolled stimulation of Ras-related functions by altered p21ras protein, locking it in the ‘on’ position for signal transduction (Adjei, 2001; Molina and Adjei, 2007).

Non-small cell lung cancers (NSCLC) represent more than 80% of lung cancers and are subgrouped in squamous cell carcinomas (SCC), adenocarcinoma (ADC) and large cell carcinoma (Travis *et al*, 2004). *K-Ras* mutations are found in 10–20% of NSCLC and have been described in approximately 30% of ADC (Ahrendt *et al*, 2001). About 92% of *K-Ras* mutations occur in codon 12 (Huncharek *et al*, 1999). *K-Ras* mutations are most closely associated with a history of cigarette smoking and are more common in women (Ahrendt *et al*, 2001; Broermann *et al*, 2002; Sugio *et al*, 2006; Tam *et al*, 2006). Oncogenic activation of K-Ras has been reported as a prognostic marker of poor outcome in NSCLC patients and *K-Ras* mutations seemed to be associated with a shorter survival in early-stage and locally advanced NSCLC (Fukuyama *et al*, 1997; Huncharek *et al*, 1999). A recent meta-analysis of *K-Ras* mutations in lung cancer showed that these mutations appeared to be associated with shorter survival in NSCLC (Mascaux *et al*, 2005). It was also suggested that *K-Ras* mutations could be predictive of chemotherapy resistance, in metastatic disease rather in adjuvant situation (Eberhard *et al*, 2005; Tsao *et al*, 2007). Although meta-analyses has indicated that *K-Ras* gene mutations are weak prognostic markers of poorer outcome in NSCLC, results from individual studies have been inconsistent (Huncharek *et al*, 1999; Mascaux *et al*, 2005; Tsao *et al*, 2007).

At first, EGFR status is a decisive molecular factor for using EGFR-targeted therapies in NSCLC (Lynch *et al*, 2004; Paez *et al*, 2004; Pao *et al*, 2004). *K-Ras* and *EGFR* mutations were shown to be mutually exclusive in lung ADC (Kosaka *et al*, 2004). Mutations in *K-Ras* are found more frequently in patients who develop disease progression upon gefinitib or erlotinib therapy (Pao *et al*, 2005). Both the time to disease progression and the median survival were significantly worse in patients with *K-Ras* mutations when they were treated with erlotinib in combination with chemotherapy compared with those receiving chemotherapy alone (Eberhard *et al*, 2005). It was suggested that one potential mechanism for primary resistance to EGFR-TKI may be the presence of *K-Ras* mutations (Massarelli *et al*, 2007). These recent advances within molecular biology may facilitate treatment selection based on potential predictive molecular markers such as *K-Ras*.

Direct sequencing is considered as the gold standard and has allowed comprehensive knowledge of mutations (Lynch *et al*, 2004; Paez *et al*, 2004; Pao *et al*, 2004, 2005; Massarelli *et al*, 2007). Direct sequencing is still widely employed to discover ‘new’ mutations. But the sensitivity of direct sequencing depends on the percentage of tumour cells in the analysed sample. A percentage of more than 50% of tumour cells is usually requested and the threshold of detection is around 25% of mutant DNA into a wild-type environment (Pao and Ladanyi, 2007; Eberhard *et al*, 2008). If a low percentage of tumour cells is present in some samples, direct sequencing could lead to false-negative results. Thus, direct sequencing considered as the gold standard could be inadequate in such cases, even in surgical specimens, resulting in differences in *K-Ras* mutations frequencies and in prognostic/predictive values of such mutations. Besides classical sequencing, *K-Ras* mutations could be detected by mutation-specific oligonucleotide hybridisation, PCR followed by restriction fragment length polymorphisms analysis, single-strand conformation polymorphisms analysis or mutant allelic-specific amplification. These procedures involve multiple steps and/or are time-consuming. Therefore, they are impracticable for routine clinical use.

To detect a minimal amount of mutant DNA in clinical samples, peptide-nucleic-acid (PNA) oligomers have been developed (Orum *et al*, 1993). PNAs are non-extendable oligonucleotides in which the ribose-phosphate backbone is replaced by 2-aminoethyl glycine units linked by amide bonds. In PNA-mediated PCR clamping, PNA oligomers suppress the amplification of the complementary sequence confined by a pair of DNA oligonucleotide primers because PNA are not substrate for DNA polymerase. The PNA-clamped probe assay is more sensitive than direct sequencing, with the ability to detect mutations in samples containing less than 1% mutant alleles (Nagai *et al*, 2005; Pao and Ladanyi, 2007). Using this method, *c-Kit* point mutations have been detected in skin biopsy samples from patients with urticaria pigmentosa (Sotlar *et al*, 2003). Such a technique has been applied to search for *K-Ras* mutations in various tumour samples (Sun *et al*, 2002; Chen *et al*, 2004; Taback *et al*, 2004; Däbritz *et al*, 2005; Luo *et al*, 2006; Miyake *et al*, 2007). This method was also used to detect *EGFR* mutations in NSCLC (Nagai *et al*, 2005; Soh *et al*, 2006; Sutani *et al*, 2006; Tanaka *et al*, 2007; Miyanaga *et al*, 2008).

As NSCLC, in particular adenocarcinoma, could often be mixed with normal cells, the aim of this study was to estimate the possibility to increase the sensitivity of the detection of *K-Ras* mutations on an EGFR-targeted naive NSCLC cohort, even in cases of low tumour cellularity and to evaluate its routine clinical usefulness. To be able to compare the results of detection of *K-Ras* mutations by a sensitive technique and by direct sequencing, we chose to work on frozen samples of NSCLC. We conducted this study to establish a one-step real-time PCR method that combines fluorescent hybridisation probes PCR without or with competing wild-type PNA 17-mer and melting curve analysis for the detection of *K-Ras* mutations at codon 12 and 13, in a cohort of 114 consecutive surgical frozen NSCLC tumour tissues.

## Materials and methods

### Patients

Anonymised frozen samples from 114 routine consecutive NSCLC patients surgically treated were obtained from the Biological Resource Center of the University Hospital of Strasbourg, in protocols approved by the institutional review board. Stage was defined as recommended (Moutain, 1997). All patients are chemo- and targeted drug naive at the time of surgery. All the patients are non-Asian. The patients included 91 men and 23 women. Only seven (6%) of the patients were never smokers. The observation period ranged from 1 to 82 months, with a median follow-up of 26 months. Patients’ characteristics are summarized in Table 1. Tumour and paired normal lung peripheral tissue samples obtained at the time of surgery, were immediately stored at −80°C. Tumours were histologically classified according to the World Health Organization guidelines and scored for differentiation (Travis *et al*, 2004). Three haematoxylin and eosin-stained sections of frozen tissues were reviewed by a pathologist to evaluate the percentage of tumour cells, at the beginning, middle and end of the frozen tissues. The percentage of tumour cells corresponds to the number of tumour cells reported to the number of all the cells (tumour and non-tumour cells) analysed on a slide. Almost half of the patients presented less than 50% of tumour cells. Histological characteristics are summarized in Table 1.

### DNA extraction from frozen lung cancer tumours

Genomic DNA was isolated using conventional techniques with QIAamp DNA kit (Qiagen, Courtaboeuf, France). To validate the percentage of tumour cells assessed by the pathologist in the frozen samples, paired tumour and normal DNA were amplified by fluorescent polymerase chain reaction (PCR) for microsatellite (MS) analysis with a panel of markers distributed among the genome on 13 chromosomes frequently altered in NSCLC (data not shown). Detailed MS and PCR conditions are available upon request.

### DNA sequencing analysis

The mutational status of *K-Ras* (codon 12,13) was performed using 40 ng of genomic DNA amplified by PCR in 25 *μ*l reaction mix containing 1.25 U Fast Start High Fidelity Taq (Roche), 0.2 mM dNTP, 1.5 mM MgCl_2_, and 0.2 *μ*m of *K-Ras* F and *K-Ras* R I primers (Table 2). After an initial denaturation (2 min at 94°C), a touch down protocol was used as follows: 10 cycles with a decreased hybridisation temperature from 61 to 58°C every two cycles. The subsequent 35 cycles were performed as follows: 50 s at 93°C, 50 s at 55°C, and 1 min 30 s at 72°C, followed by a final extension period of 10 min at 72°C. All PCR products were verified by electrophoresis on agarose gels. After purification (Microcon-PCR Filter Unit, Millipore, Paris, France), the PCR products were sequenced (Big Dye Terminator v1.1 Cycle sequencing kit, Applied Biosystems, Forster City, CA, USA) and analysed on ABI PRISM 3100 Genetic Analyser (Applied Biosystems). The GB sequence of human *K-Ras* (L00045) was used as a reference for sequence analysis (Seqscape v2.5, Applied Biosystems). All sequencing reactions were performed in both forward and reverse directions, and all mutations were confirmed by sequencing a second independent PCR product. As expected, by direct sequencing technique for K-Ras mutation analysis, *K-Ras* mutations were reproducibly detectable at a dilution of 25% of mutated DNA into normal DNA (data not shown). All the mutated *K-Ras* DNA were also sequenced for *EGFR* mutation analysis as described earlier (Beau-Faller *et al*, 2008).

### Real-time PCR and melting curve analysis, clamped-probe assay

**Figure 1:**
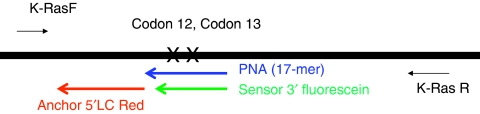
Schematic representation using PNA-mediated PCR for detection of mutant *K-Ras*. Relative positions of PCR primers (*K-Ras* F, *K-Ras* R), wild-type hybridisation probes (Anchor, Sensor) and peptide-nucleic-acid (PNA) for K-Ras mutations detection at codons 12 and 13.

Primers were chosen to amplify a specific 243-bp genomic fragment from *K-Ras* codon 12 and 13. Hybridisation FRET probes were designed complementary to wild-type sequence of codon 12 and 13. The anchor probe was 5′-labeled with LC-Red 640 and 3′ phosphorylated, and the sensor probe was 3′-labeled with fluorescein. The PNA oligomer covered codons 10–14 wild-type sequence. Sequences of primers (Eurogentec, Liège, Belgium), probes (Timolbiol, Berlin, Germany), and 17 mer-PNA (Eurogentec, Liège, Belgium) are listed in Table 2. They are represented in Figure 1. Real-time PCR was performed with 20 ng of genomic DNA, in a final volume of 20 *μ*l containing Light Cycler Fast Start DNA Master Hyb Probe (Roche Diagnostics, Mannheim, Germany), 3 mM MgCl_2_, 0.4 *μ*M of each primers *K-Ras*, 0.4 *μ*M of *K-Ras* hybridisation sensor probe and 0.2 *μ*M of *K-Ras* hybridisation anchor probe, without or with 0.1 *μ*M PNA oligomer. After the initial denaturation step at 95°C for 10 min, a touch down amplification was performed consisting of a denaturation at 95°C for 10, 7 s at 76°C, then 10 cycles with touchdown annealing for 15 s from 65 to 55°C (decreasing 2°C/two cycles) and elongation at 65°C for 20 s. This step was followed by 28 cycles: 95°C for 10 s, 76°C for 7 s, 50°C for 15 s, 65°C for 20 s. Melting curve analysis was performed by increasing temperature from 40 to 95°C with a transition rate of 0.25°Cs^−1^. Fluorescence data were analysed using the Light Cycler software (software version 3.5, Roche Diagnostics). Mutation analysis for each tumour sample was performed at least two times. PNA–PCR products from all samples that gave positive results by the clamped-probe assay was sequenced to confirm and precise the type of *K-Ras* mutation.

### Controls

**Figure 2:**
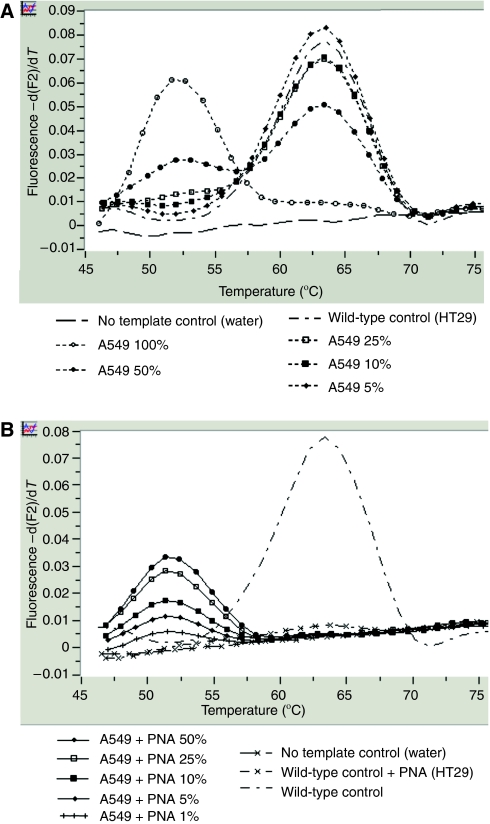
Serial dilution experiments showing the sensitivity of the alternative techniques for *K-Ras* mutation detection: hybridisation assay without PNA (**A**) and PNA-clamped probe assay (**B**). DNA from A549 (lung adenocarcinoma) cell line, which harbours an homozygous *K-Ras* mutation (G12S), was diluted into DNA from a wild-type *K-Ras* HT29 cell line.

DNA from a lung cancer cell line A549 with a homozygous *K-Ras* codon 12 mutation (c.34G>A, G12S) was used for homozygous *K-Ras* mutation positive control. One negative control (DNA from colon cancer cell line HT29 of colon carcinoma with wild-type *K-Ras* sequence) and a no template negative control (as control for contamination) were processed in parallel. We evaluate the sensitivity of the developed alternative methods by serially diluting DNA from A549 mutated *K-Ras* cell line in the DNA of wild-type *K-Ras* HT29 cell line (Figure 2).

### Statistical analysis

A total number of patients to be tested by PNA–PCR clamp was decided to be more than 100 patients because about 15–20% of NSCLC patients were reported to have *K-Ras* mutations in previous articles (Mascaux *et al*, 2005). Statistical analysis using *χ*^2^ test or exact test (type Fisher's test) if appropriate with exact *P*-values was used to compare the qualitative data. Where appropriate, continuous variables were categorized before analysis. The date of point was 30 April 2008. The Kaplan–Meier method was used to estimate the probability of event-free survival and the log-rank test to detect the difference in survival curves. Cox's proportional hazard models were used to determine the impact of patient characteristics on event-free survival. All statistical calculations were performed with the Statistical Package for the Social Science (SPSS) (number 15.0) statistical software.

## Results

### Sequence analysis of NSCLC samples

We first analysed the 114 routine samples by direct sequencing for *K-Ras* mutation detection. By this technique, 11 (10%) of the 114 cases showed a *K-Ras* mutation at codon 12 or 13, represented by five mutations G12C, two mutations G12D, two mutations G12V, one mutation G12R and one mutation G12A (Tables 3 and 4).

### Determination of the sensitivity of the hybridisation-probe assay and clamped-probe assay

The tumour cell count was lower than 50% in 52 patients, and among them lower than 25% in 15 patients (Table 1). To further improve the detection of *K-Ras* mutations in comparison with direct sequencing, we developed real-time PCR with wild-type hybridisation probes without or with PNA-mediated clamped PCR. We assessed the sensitivity of these assays by testing homozygous G12S *K-Ras* mutated A549 cell line DNA serially diluted into DNA from wild-type *K-Ras* HT29 cell line DNA. By hybridisation-probe assay, the mutations were reproducibly detectable at a dilution of 10% of mutated DNA into wild-type DNA and by the clamped-probe assay, at a dilution of 1% of mutated DNA into wild-type DNA (Figure 2). Indeed, in our conditions, addition of PNA allows to increase the threshold detection of *K-Ras* mutation by a magnitude of 10.

### Hydridisation-probe and clamped-probe assays for detecting *K-Ras* mutations. Comparison with direct sequencing

**Figure 3:**
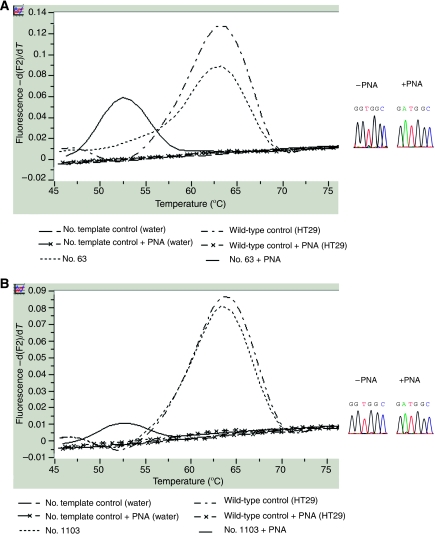
Representative melting curves obtained by hybridisation and clamped-probe assays. On the right side, paired electropherograms obtained by sequencing of a PCR product, without and with PNA. (**A**) *K-Ras* mutation (G12D) diagnosed by the two alternative techniques: hybridisation probe and clamped-PCR assays (No. 63). (**B**) *K-Ras* mutation (G12D) only diagnosed by the most sensitive technique: clamped-PCR (No. 1103).

All the 114 patients were then analyzed for *K-Ras* mutations by hybridisation-probe assay and clamped-probe assay (Table 2). As expected, the 11out of 114 (10%) tumours with a *K-Ras* mutation at codon 12 or 13 detected by direct sequencing, were also detected by hybridisation-probe/clamped-probe assays. However, five tumours with wild-type *K-Ras* assessed by direct sequencing presented *K-Ras* mutations when using the hybridisation-probe assay. Remarkably, five other tumours with wild-type *K-Ras* assessed by direct sequencing and by the hybridisation-probe assay presented *K-Ras* mutations when using clamped-probe assay alone. As expected, all the positive samples detected by hybridisation-probe assay were also positive for clamped-probe assay. Thus, these sensitive techniques allowed an increase in detection of *K-Ras* mutations from 11 out of 114 patients (10%) by direct sequencing to 21 out of 114 (18%) (*P*<0.005). To confirm the mutations in the 10 tumours only detected by these sensitive techniques and to identify precisely the type of *K-Ras* mutation, all PNA–PCR products were directly sequenced for codon 12 and 13 of *K-Ras*. We identified five mutations G12C, three mutations G12D, one mutation G12S and one mutation G13C with hybridisation-probe/clamped-probe assays (Tables 3 and 4, Figure 3).

Among the *K-Ras* mutated tumours diagnosed only by hybridisation/PNA techniques, five had a percentage of tumour cells effectively lower than required for classical sequencing. The tumour cellularity of these tumours was 15% (one patient), 10% (one patient) and less than 10% (three patients). *K-Ras* mutations in two of these tumours were detected by hybridisation-probe assay and three others by clamped-probe assay only. In another hand, five *K-Ras* mutated tumours diagnosed only by hybridisation/PNA techniques, presented a percentage of tumour cells usually sufficient for direct sequencing, suggesting the presence of mutated tumour subclones.

### Clinical data and *K-Ras* mutations detected by the different methods

**Figure 4:**
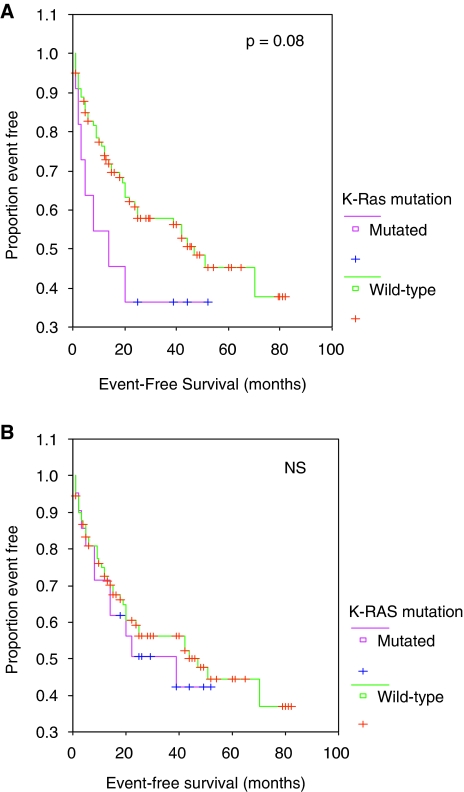
Kaplan–Meier curves for event-free survival (months) of 114 NSCLC tumours. (**A**) Stratified according to *K-Ras* mutation analysed by direct sequencing (median EFS 14 months *vs* 47 months, HR=1.57, CI 95%: 0.77–3.84). (**B**) Stratified according to *K-Ras* mutation analysed by PCR clamp assay (median EFS 16 months *vs* 47 months, HR=1.19, CI 95%: 0.61–2.32).

Then, we asked for the clinical usefulness of *K-Ras* mutations detected by these sensitive techniques. Our cohort of 114 surgically treated NSCLC patients were represented by 55 ADC and 59 SCC (Table 1). The 11 *K-Ras* mutations detected by direct sequencing were found in seven ADC (two with BAC features) and in four SCC (Tables 1 and 4). The 10 additional *K-Ras* mutations detected by hybridisation/PNA methods were observed in eight ADC and in two SCC. Overall, the 21 *K-Ras* mutated patients, 16 males and five females, 15 ADC and six SCC, have tumours from all stages and from current/former smokers. In adenocarcinoma, *K-Ras* mutation frequency increased from 7 out of 55 (13%) by direct sequencing to 15 (27%) by clamped-PCR (*P*<0.005). If there is no correlation between *K-Ras* mutation frequency detected by direct sequencing and histological subtypes, a correlation was found between *K-Ras* mutation frequency detected by hybridisation/PNA methods and ADC histological subtype (*P*=0.01). Survival analysis showed that the presence of *K-Ras* mutations diagnosed by direct sequencing are a weak prognostic marker of poorer outcome (median EFS 14 months *vs* 47 months, HR=1.57, CI 95%: 0.77–3.84, *P*=0.08). By contrast, when taking into account all the *K-Ras* mutations detected by the three techniques, our results failed to confirm *K-Ras* oncogenic activation as a significant marker of poor prognosis after surgery for NSCLC (median EFS 16 months *vs* 47 months, HR=1.19, CI 95%: 0.61–2.32, NS) (Figure 4).

## Discussion

Surgical tumour specimens of NSCLC may contain a lot of normal/inflammatory cells. The presence of a high percentage of normal/inflammatory cells could lead to ‘false-negative’ results when mutations were detected by direct sequencing. A recent commentary synthetically addresses the question of the ideal method for *EGFR* mutation testing in lung cancer (Pao and Ladanyi, 2007), but no such data are available for *K-Ras*. We developed a quick, cheap and sensitive method for detecting *K-Ras* mutations from routine surgical specimens of NSCLC without removing contaminating normal cells by macro/micro dissection.

PNA–PCR clamp method can rapidly (within 2 h) detect *K-Ras* mutations using a low quantity of DNA. By contrast to other PNA studies, we used wild-type fluorescent-labelled hybridisation probes allowing rapid and high-sensitive detection of all the *K-Ras* mutations (Sun *et al*, 2002; Chen *et al*, 2004; Taback *et al*, 2004; Däbritz *et al*, 2005; Luo *et al*, 2006; Miyake *et al*, 2007). Only one pair of primers and one pair of probes are required to detect all possible mutations in codons 12 and 13 of the *K-Ras* gene. The detected fluorescence signal corresponds to the amplified mutant DNA and can be analysed by a subsequent melting curve analysis. If requested, the PNA–PCR products are available to precisely identify the mutated nucleotide. All these advantages greatly simplify the manipulating procedure.

The sensitivity of direct sequencing depends on the percentage of tumour cells in the analysed sample, generally up to 50% tumour cells is requested with a detection threshold of 25% mutant DNA into a wild-type environment (Pao and Ladanyi, 2007; Eberhard *et al*, 2008). The sensitivity of our assays is 10 and 1% of mutant DNA for hybridisation-probe assay and for clamped-probe assay, respectively, and might correspond to a more sensitive test, applicable for routine purposes without macro or micro dissection. Another new rapid method for mutated *EGFR* and *K-Ras* detection by high resolution melting analysis (HRM) has been recently reported (Do *et al*, 2008). Even HRM is a promising method, it can be compromised by a low proportion of tumour cells in the analysed sample and by the difficulty to detect homozygous mutations, as the sensitivity of *K-Ras* mutations detection was only 5–10%. Among the 10 new *K-Ras* mutated patients detected by these most sensitive methods, half presented a percentage of tumours cells under the level usually required for direct sequencing. Thus, even in a surgical cohort of NSCLC tumour samples, there is a risk of false-negative *K-Ras* mutated patients when they were analysed by direct sequencing. The rapidity and sensitivity of our sensitive technique could lead us to propose this test for routine *K-Ras* mutation detection, that is, bronchial biopsies in NSCLC.

In our study, the 10 new *K-Ras*-mutated cases presented clinical/biological characteristics usually associated with *K-Ras* mutations in NSCLC (Ahrendt *et al*, 2001; Broermann *et al*, 2002; Sugio *et al*, 2006; Tam *et al*, 2006). Remarkably, none of the five new *K-Ras* mutated patients with more than 50% of tumour cells, presented an *EGFR* mutation (exon 18, 19, 20, 21) (data not shown). Interestingly, if *K-Ras* mutations diagnosed by direct sequencing appeared to be a prognostic marker of poorer outcome in our cohort of surgically treated NSCLC patients, this tendency is not more observed when we considered *K-Ras* mutations diagnosed by the two other techniques (hybridisation probe alone, or with clamped-PCR). Our survival analysis is nevertheless limited by the relatively short follow-up with fewer than half of the patients having relapsed or died. Accordingly, a recent study (JBR.10) showed that mutations of *K-Ras* gene were neither prognostic for survival nor predictive of a differential benefit from adjuvant chemotherapy in stage IB and II NSCLC (Tsao *et al*, 2007). In this study, the tumour cellularity was enriched by microdissection, and using allelic-specific oligonucleotide hybridisation, they failed to confirm *K-Ras* oncogenic activation as a significant marker of poor prognosis after surgery for NSCLC. The lack of prognostic value of all the *K-Ras* mutated cases could be explained by the fact that in cases of mutation detected by sensitive techniques, the real percentage of *K-Ras* mutated cells could be too low, under a threshold with immediate clinical significance. Further studies are required to precisely determinate the significant prognostic threshold of *K-Ras* mutated cells.

In our study, half of the new *K-Ras* mutated cases diagnosed only by sensitive techniques, presented a percentage of tumour cells above the threshold usually required for direct sequencing. These results suggest the presence of mutated subclones in the tumour. In fact, direct sequencing could fail to detect mutated subclones (Pao and Ladanyi, 2007). Some *EGFR*-mutated subclones were already detected in NSCLC when *EGFR* T790M mutations have been analysed with sensitive techniques (Inukai *et al*, 2006). It was suggested that the possible presence of such mutations at a low frequency in NSCLC tumours before EGFR-targeted therapy might affect the tumour response or the event-free survival after targeted treatments. Recently, *K-Ras* mutations detected by allelic discrimination on tumour DNA, have been demonstrated to be an independent prognostic factor in patients with advanced colorectal cancer treated with cetuximab (Lièvre *et al*, 2008). To our knowledge, no predictive studies have been realised to identify *K-Ras* mutated subclones in NSCLC, but the presence of such mutated subclones could explain some cases of secondary EGFR-targeted therapy resistance. Therefore, it would be important to evaluate the predictive value of such *K-Ras* mutated tumour subclones, particularly in stabilised or progressive cases treated by targeted EGFR therapy.

Consequently, the percentage of tumour cells in the analyzed sample as well as the threshold of mutation detection of the currently molecular test appeared to be two essential data to better understand the significance (i.e., prognostic or predictive factor) of the detected mutation. Indeed, in our cohort of surgical specimen, 53 (46.5%) of our routine consecutive patients presented a percentage of tumour cells under the value (50%) classically required for direct sequencing; and in addition, some patients with a percentage of tumour cells sufficient for direct sequencing, appeared to have *K-Ras* mutations at a low frequency. In the first type of tumours, a sensitive technique will detect low level of *K-Ras* mutations with finally a number of K-Ras mutated tumour cells which could have clinical usefulness; in the second type of tumours, sensitive technique will detect *K-Ras*-mutated subclones of which clinical usefulness has still to be explored.

In conclusion, our study allows us to propose an easy and sensitive method for rapid analysis of *K-Ras* mutations in NSCLC tumours. This sensitive technique could be helpful for specimens of lung cancer without removing contaminating normal cells, mostly in the setting of NSCLC, in which diagnoses are often based on bronchial biopsies or cytologic specimens. The prognostic value of such sensitive detected *K-Ras* mutations when tacking into account the tumour cellularity, has still to be evaluated on an increased number of surgically treated lung cancer patients, taking into account the frequency of mutated allele in the sample depending on the tumour cellularity. Furthermore, predictive value of low represented *K-Ras* mutations need to be assessed in lung cancer patients treated by targeted anti-EGFR treatments, to optimise such treatments in NSCLC patients.

## Figures and Tables

**Table 1:** Patients’characteristics

	**All NSCLC**	**ADC/BAC**	**SCC**
All	114	55^a^	59
Age (years): mean (s.d.)	61 (10)	61 (10)	61.5 (10)
			
*Gender*			
Men	91 (80%)	37 (68%)	54 (91.5%)
Women	23 (20%)	18 (33%)	5 (8.5%)
Current/former smoker	106 (93%)	47 (87%)	59 (100%)
Never-smoker	7 (6%)	7 (13%)	0 (0%)
			
*Stage pTNM*			
I–IIIA	99 (87%)	45 (82%)	54 (91.5%)
IIIB–IV^b^	15 (13%)	10 (18%)	5 (8.5%)
			
*Histologic differentiation*			
Well/moderate	74 (65%)	28 (57%)	46 (80%)
Poor	32 (28%)	21 (43%)	11 (19%)
			
*Tumour cell count*			
<50%	52 (46%)	28 (51%)	24 (41%)
⩾50%	62 (54%)	27 (49%)	35 (59%)
			
*Lung cancer relapse*			
Yes	52 (45%)	28 (54%)	23 (39%)
No	60 (53%)	24 (46%)	36 (61%)
			
*Lung cancer death*			
Yes	53 (46.5%)	28 (53%)	25 (42%)
No	59 (52%)	25 (47%)	34 (58%)

NSCLC=non-small cell lung cancer; ADC=adenocarcinoma; BAC=bronchioloalveolar carcinoma; SCC=squamous cell carcinoma.

Clinical and pathological features of 114 NSCLC patients with NSCLC, classified by histological subtypes.

a Of whom eight BAC features.

b 7 stages IV with unique metastasis.

**Table 2:** DNA sequences of primers, PNA oligomer, and probes for detecting *K-Ras* mutations

**Name**	**DNA (5′–3′) or PNA (NH_2_-CONH_2_) sequence^a^**	**Position,^b^ nt**
K-Ras F^c^	GGAGTATTTGATAGTGTATTAACCT	9–33
K-Ras R I	AGAATGGTCCTGCACC	251–236
K-Ras R II	GTCCTGCACCAGTAATATGC	244–226
Anchor	LC-Red 640-ACTACCACA AGTTTATATTCAGTCATTTTCAGCAGG-Ph	121–86
Sensor	CCTAC**GCCACC**AGCTC-Flu	138–124
PNA	CCTAC**GCCACC**AGCTCC	138–122

a The 3′ end of the anchor probe was phosphorylated to prevent probe elongation by Taq polymerase during PCR. Underlined, nucleotide complementary to wild-type bases of codon 12 and 13.

b The base numbering is according to GenBank accession no L00045.

c F=forward; R=reverse; Flu=fluorescein; LC-Red=LightCycler-Red; Ph=phosphorylated; R I: K-Ras reverse primer used for Light-cycler analysis and classical sequencing; R II: K-Ras reverse primer used for sequencing of PNA–PCR products.

**Table 3:** Types of *K-Ras* mutations found in 114 NSCLC tumours

**(Codon)mutation^a^ (aminoacid change)**	**Sequencing (%)^b^**	**Hybrisation (%)^b^**	**PNA (%)^b^**
(12) GGT → AGT (Gly → Ser)	0 (0)	0 (0)	1 (5)
(12) GGT → CGT (Gly → Arg)	1 (9)	1 (6)	1 (5)
(12) GGT → TGT (Gly → Cys)	5 (45.5)	8 (50)	10 (48)
(12) GGT → GAT (Gly → Asp)	2 (18)	3 (19)	5 (24)
(12) GGT → GCT (Gly → Ala)	1 (9)	1 (6)	1 (5)
(12) GGT → GTT (Gly → Val)	2 (18)	2 (12.5)	2 (9.5)
(13) GGC → TGC (Gly → Cys)	0 (0)	1 (6)	1 (5)
*K-Ras* mutation	11	16	21
Wild-type	103	98	93
Total	114	114	114

a Altered bases are underlined.

b (%) Percentage of mutation type reported to mutated cases.

**Table 4:** Clinical and biological characteristics of *K-Ras* NSCLC-mutated patients

**No**	**Sex**	**Age (years)**	**Histological type**	**Tumour cells count (%)**	**Stage**	**Relapse**	**EFS (months)**	**Technique using for *K-Ras* mutation detection**	**Type of *K-Ras* mutation**
77	M	51	ADC	90	IIB	Yes	2	SQC	G12C
108	F	52	ADC	70	IIIA	Yes	14	SQC	G12C
118	M	68	SCC	60	IB	No	25	SQC	G12D
24	M	58	ADC	60	IB	No	39	SQC	G12C
1109	M	60	ADC-BAC	50	IIB	No	52	SQC	G12A
50	M	54	SCC	50	IA	Yes	20	SQC	G12D
1111	M	66	SCC	50	IA	Yes	8	SQC	G12V
1124	F	44	ADC	50	IIIA	Yes	5	SQC	G12R
1104	M	70	ADC-BAC	40	IIIB	No	44	SQC	G12C
1118	F	53	ADC	40	IV	Yes	1	SQC	G12C
9915	M	60	SCC	40	IIB	Yes	3	SQC	G12V
22013	F	60	SCC	90	IB	No	18	HYB	G12C
52	M	44	ADC	70	IIIA	Yes	22	HYB	G12C
63	F	44	ADC	50	IA	No	49	HYB	G12D
91	M	67	ADC	15	IB	No	18	HYB	G12C
1126	M	74	ADC	1	IA	No	29	HYB	G13C
1116	M	55	ADC	50	IIB	No	26	PNA	G12S
22006	M	56	ADC	50	IIIA	Yes	14	PNA	G12C
1103	M	76	ADC	10	IIIA	Yes	8	PNA	G12D
121	M	45	SCC	1	IB	No	49	PNA	G12C
102	M	56	ADC	1	IIIA	Yes	39	PNA	G12D

ADC=adenocarcinoma; BAC=bronchioloalveolar carcinoma; SCC=squamous cell carcinoma EFS=event-free survival; SQC=results obtained by direct sequencing; HYB=results obtained by hybridisation probe assay; PNA=results obtained by clamped-probe assay.
